# Greig Cephalopolysyndactyly Contiguous Gene Syndrome: Case Report and Literature Review

**DOI:** 10.3390/genes12111674

**Published:** 2021-10-23

**Authors:** Kinga Kozma, Marius Bembea, Claudia M. Jurca, Mihai Ioana, Ioana Streață, Simona Ş. Şoşoi, Andrei Pirvu, Codruța D. Petchesi, Ariana Szilágyi, Cristian N. Sava, Alexandru Jurca, Anikó Ujfalusi, Zsuzsanna Szűcs, Katalin Szakszon

**Affiliations:** 1 Faculty of Medicine and Pharmacy, University of Oradea, 410073 Oradea, Romania; bembea13@yahoo.com (M.B.); claudiajurca70@yahoo.com (C.M.J.); szilagyi.ariana@gmail.com (A.S.); cristian.sava2004@gmail.com (C.N.S.); alexjurca@yahoo.co.uk (A.J.); 2 Regional Center of Medical Genetics Bihor, 410445 Oradea, Romania; 3 Municipal Clinical Hospital “Dr. Gavril Curteanu”, 410469 Oradea, Romania; 4 Regional Center of Medical Genetics Dolj, 200349 Craiova, Romania; mihai.ioana@umfcv.ro (M.I.); ioana.streata@yahoo.com (I.S.); simona.sosoi@umfcv.ro (S.Ş.Ş.); andrei.pirvu9@gmail.com (A.P.); 5 Human Genomics Laboratory, Faculty of Medicine, University of Medicine and Pharmacy Craiova, 200642 Craiova, Romania; 6 Division of Clinical Genetics, Faculty of Medicine, Departament of Laboratory Medicine, University of Debrecen, 4032 Debrecen, Hungary; ujfalusi.aniko@med.unideb.hu (A.U.); szucs.zsuzsanna@med.unideb.hu (Z.S.); 7 Faculty of Medicine, Departament of Pediatrics, University of Debrecen, 4032 Debrecen, Hungary; szakszon.katalin@gmail.com

**Keywords:** Greig cephalopolysyndactyly, Greig cephalopolysyndactyly contiguous gene syndrome, array-CGH, structural chromosomal anomalies, deletion 7p

## Abstract

Greig cephalopolysyndactyly syndrome (GCPS) is a rare genetic disorder (about 200 cases reported), characterized by macrocephaly, hypertelorism, and polysyndactyly. Most of the reported GCPS cases are the results of heterozygous loss of function mutations affecting the *GLI3* gene (OMIM# 175700), while a small proportion of cases arise from large deletions on chromosome 7p14 encompassing the *GLI3* gene. To our knowledge, only 6 patients have been reported to have a deletion with an exact size (given by genomic coordinates) and a gene content larger than 1 Mb involving the *GLI3* gene. This report presents a patient with Greig cephalopolysyndactyly contiguous gene syndrome (GCP-CGS) diagnosed with a large, 18 Mb deletion on chromosome 7p14.2-p11.2. Similar cases are reviewed in the literature for a more accurate comparison between genotype and phenotype.

## 1. Introduction

Greig cephalopolysyndactyly syndrome (GCPS) is a rare genetic disorder (~200 cases reported), characterized by macrocephaly, hypertelorism, polysyndactyly and most commonly normal intelligence [1,2,3,4].

Both small-scale (missense, nonsense, splicing variants, small deletions, small insertions, and indels) and large-scale alterations (gross deletions, gross insertions, and translocations) affecting the *GLI3* gene (~300 kb), located on chromosome 7p14.1 can cause GCPS (MIM# 175700) [5] in an autosomal dominant mode of inheritance [1,2,6,7,8].

Cases with a chromosomal microdeletion encompassing *GLI3* (deletions larger than 1 Mb) are referred to as GPS-CGS, and they typically present with a more complex neurobehavioral phenotype including intellectual disability, severe motor retardation and neurological symptoms [2,6].

In this study, we describe a child of non-consanguineous parents with severe developmental disability diagnosed with a large interstitial deletion of chromosome 7p, using array comparative genomic hybridization. The molecular karyotype revealed a deletion of 18 Mb on chromosome 7p14.2-p11.2, which is the largest chromosomal deletion described in the related literature so far [1,2,3,4,5,6,7,8,9,10,11,12,13,14,15,16,17,18,19].

## 2. Materials and Methods

### 2.1. Patient

A 3-year-old boy was referred to our Genetics Department of Municipal Clinical Hospital “Dr. Gavril Curteanu” Oradea, Romania, due to psychomotor developmental delay and multiple congenital anomalies. He is the second child of healthy and unrelated parents, whose first child is a healthy girl.

The patient was born from an uneventful pregnancy as the second child of the parents, in the 34th week, via vaginal delivery. Macrosomia (the birth weight was 3030 g, 97th percentile and length 48 cm, 90th percentile), macrocephaly (OFC was 36 cm, 1 cm > 97th percentile), and congenital malformations of the face and limbs were noted at birth. Perinatal hypoxia was suspected based on the Apgar scores (5/1, 7/5, and 8/10).

### 2.2. Neuroimaging

Cranial ultrasound at birth and magnetic resonance imaging (MRI) was performed at 1 year and at 3 years.

### 2.3. Classical and Molecular Cytogenetic Investigations

An informed consent was obtained from the patient’s parents as legal representatives, to collect blood, take pictures, and access medical data.

Karyotyping was performed at the Clinical Genetics Center of the University of Debrecen, Hungary, from peripheral blood using the G-banding technique, with 500 bands, 15 metaphases were analyzed. Image analysis was done using CytoVision Software.

Molecular cytogenetic analyses were performed at the Regional Center of Medical Genetics Dolj (Romania). Genomic DNA was extracted from peripheral venous blood using Wizard^®^ Genomic DNA Purification Kit (Promega, Madison, WI, USA), following the manufacturer’s protocol. DNA concentration and quality were measured using a spectrophotometer (Eppendorf Biophotometer). A total of 0.5 µg of genomic DNA was used for array comparative genomic hybridization (aCGH) analysis following the manufacturer’s protocol. It was performed using a microarray slide with 120,000 oligonucleotide and 60,000 SNP probes (180K) covering the entire human genome with an average spatial resolution of 25 kb DNA (G4890A, ID design: 029830_20100921, Genom hg38 build; Agilent). The post-hybridization data were obtained using the Feature Extraction programme, and their subsequent analysis was performed with the CytoGenomics programme, Agilent.

## 3. Results

### 3.1. Morphological Evaluation of the Patient

**Figure 1:**
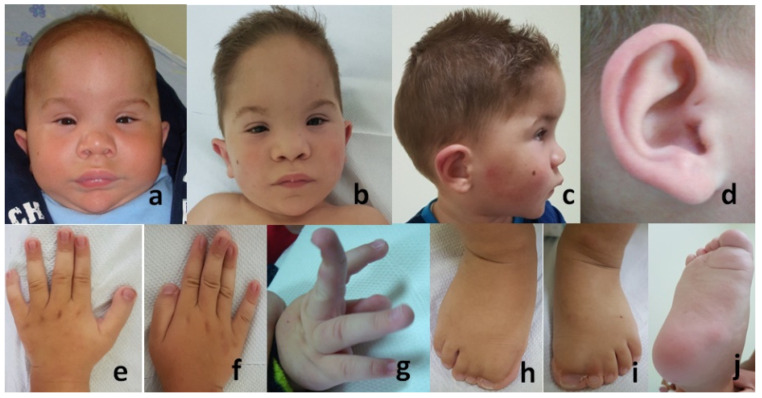
(**a**–**d**) shows cranio-facial features of the patient: macrocephaly at the age of 1 year (**a**), and macro-acrocephaly at 3 years (**b**,**c**); prominent high anterior hairline with right frontal upsweep; sparse eyebrows, hypertelorism, telecanthus, and upslanted palpebral fissures; convergent strabism; depressed and wide nasal bridge, anteverted nares, and broad nasal base with broad and high inserted columella; long and broad philtrum, and deep naso-labial fold; thick upper lip; pointed chin with a horizontal deep crease; pigmented nevus on the right cheek (**a**–**c**); low inserted ears with out-sticking upper one-third of the pinnae, and a pit on the crus of the helix (**c**,**d**). (**e**–**j**) Digital anomalies of the hands and feet: squared broad thumbs (**e**,**f**); joint laxity with dorsiflexion of the distal phalanges (**f**,**g**); broad hallux with cutaneous complete syndactyly of interdigital spaces I–II and II–III on both feet; bilateral ped planus (**h**,**i**) and deep horizontal cutaneous skin crease at the base of the hallux (**j**).

When he was one year old, macrocephaly (OFC: 48.5 cm, 95th percentile) was observed (Figure 1a) and at the age of three, macro-acrocephaly (OFC: 53 cm, 1 cm > 95th percentile) was seen (Figure 1b,c). Observations included prominent metopic suture and high anterior hairline with right frontal upsweep; sparse eyebrows, hypertelorism, upslanted telecanthus, and palpebral fissures; convergent strabism; depressed and wide nasal bridge, anteverted nares, broad nasal base, and broad and high inserted columella; deep naso-labial creases; long and broad philtrum, and thick upper lip; microdontia; pointed chin with a horizontal deep crease; pigmented nevus on the right cheek (Figure 1a–c); low inserted ears with out-sticking upper one-third, and a pit on the crus of the helix (Figure 1c,d).

Digital anomalies of the hands and feet (Figure 1e–j) included squared, broad thumbs (e,f); joint laxity with dorsiflexion of the distal phalanges (f,g); post-operative scar after surgical correction of preaxial polydactyly and broad hallux with cutaneous complete syndactyly of interdigital spaces I–II and II–III on both feet; bilateral pes planus (h,i); and deep horizontal skin cutaneous crease at the base of the hallux (j).

Additional anomalies were: congenital stridor (almost disappeared at the age of 3 years), small umbilical hernia, postoperative scar after surgical intervention for right cryptorchidism (at age 16 month), and a retractile left testicle.

Severe cognitive and motor delays were observed: head control was achieved at 6–7 months, sitting at 2 years, independent walking has not yet been achieved, and speech is limited to pronouncing a few syllables. The Mental Scale (Bayley II) score at 3 years of age was equivalent to 8 months.

Growth was assessed based on the Fenton Growth chart for preterm infants [20] and the Longitudinal Child Growth Standards of the Central Statistics Office of Hungary, by Joubert, Darvay and Ágfalvi, 2006 [21].

Morphological features are described according to the Human Malformation Termi-nology [22,23,24,25].

### 3.2. Neuroimaging

The cranial ultrasound at birth was not informative and the cranial MRI at 1 year and at 3 years showed hypoplasia of the corpus callosum, stenosis of the cerebral aqueduct, and mild supratentorial ventriculomegaly.

### 3.3. Conventional and Molecular Cytogenetic Investigations

The result of the karyotype and the aCGH analysis are described in Table 1.

The clinical diagnosis of GCPS was made immediately after birth based on the characteristic phenotype suggestive of the syndrome.

Therapeutic interventions were focusing on correcting the digital anomalies to improve the patient’s grasping manipulation and walking skills, as well as to improve cognitive functions by introducing continuous cognitive and kinetic stimulation.

The clinical course during the 6-year follow-up suggested a stationary condition regarding somatic development, which at the age of 6 years converged to normal, with weight being 22 kg (50–75th percentile), height 116 cm (50th percentile), and head circumference 53 cm (90–95th percentile). Regarding the psychomotor development, there was a mild improvement: at the age of 6 years, he is more active, his ability to cooperate gradually improves and his speech comprehension develops in terms of simple, often repetitive stimulances; vocalization is more monosyllabic; he enjoys listening to rhymes and music; the Mental Scale (Bayley II) score equal to that of a 15 month-old toddler; his right hand is more skillful, he inserts a few geometric elements, shows some of his body parts, climbs, kneels, clings, sometimes gets up, and steps with support by one hand.

## 4. Discussion

The diagnosis of GCPS was suggested immediately after birth based on the characteristic clinical signs of the syndrome and was confirmed by classical and molecular karyotyping.

The G-banded karyotype was 46,XY,del(7)(p13p15).

Molecular karyotype analysis (array-CGH) revealed an 18.37 Mb interstitial deletion on the short arm of chromosome 7. Deletion breakpoints are located between p14.2 and p11.2 bands (35830920-54201451). The OMIM genes located in the deleted region are described in Table 1. The analysis of haploinsufficiency described a difference of 330 kb, which is due to the different localization of the CNV and SNP probes.

Of the 205 genes included in the deleted segment, 22 were phenotype associated OMIM genes. Nine genes: *GLI3* (Gli-Kruppel Family Member 3; OMIM# 16540), *ANLN* (Actin-Binding Protein Anillin; OMIM# 616027), *POU6F2* (Pou Domain, Class 6, Transcription2; OMIM# 609062), *GCK* (Glucokinase; OMIM#138079), *CCM2* (Scaffold Protein; OMIM# 607929), *IKZF1* (Ikaros Family Zinc Finger 1; OMIM# 603023), *CDK13* (Cyclin-Dependent Kinase 13; OMIM# 603309), *CAMK2B* (Calcium/Calmodulin-Dependent Protein Kinase Ii-β; OMIM# 607707), and *RALA* (Ras-Like Protooncogene A; OMIM# 179550) are known to be responsible for autosomal dominant mendelian disorders, of which *GLI3* is the gene causing GCPS. Loss of function mutations of *GLI3* determine the classic phenotype with craniofacial dysmorphism (macrocephaly, hypertelorism) and polysyndactyly; while gain of function mutations determine the Pallister-Hall syndrome (MIM# 146510) [3,8,10,13].

Mutations of the *ANLN* gene have been linked to functional or structural renal anomalies; those of the *POU6F2* gene are associated with susceptibility to Wilms tumor (MIM# 601583); mutations of the *GCK* gene cause MODY II diabetes; pathogenic variants of the *CCM2* gene cause cavernous cerebral malformation; and those of the *IKZF1* gene result in immunodefficiency, providing a reason for close follow-up and screening for comorbidities in our patient.

Five years after the diagnosis, we have reevaluated our case; we updated the OMIM genes involved in the deleted segment and we found three recently phenotype-associated, autosomal dominant OMIM genes: *CDK13*, *CAMK2B*, and *RALA*. Only the *RALA* gene has been mentioned in four cases before (Jennifer J Johnston et al.) [6,7], but at that time (2003 and 2007) it had no known associated phenotype.

The *CDK13* and *CAMK2B* genes have been recently annotated OMIM genes, related to syndromic neurodevelopmental disorders (congenital heart defects, dysmorphic facial features, intellectual developmental disorder associated to MIM# 617360, mental retardation, and autosomal dominant 54 associated to MIM# 617799) [26,27,28,29,30,31,32,33].

*CDK13* codes for a member of the cyclin-dependent serine/threonine protein kinase (STK) family, which phosphorylate targets, such as RNA polymerase II-involved in extracellular and growth signaling [33]. The majority of mutations in CDK13 are missense mutations, occurring within the protein kinase domain leading to significant loss of catalytic activity. Hamilton et al. (2018) postulated their dominant-negative effect [26]. Genotype–phenotype studies suggested a trend toward milder phenotypes in patients with mutations predicted to cause haploinsufficiency of *CDK13* [30].

The *CAMK2B* gene encodes a subunit of calcium/calmodulin-dependent protein kinase II (CaM kinase II, CAMK2), a multifunctional serine/threonine kinase that has critical roles in the induction of hippocampal long-term potentiation and, as such, in synaptic plasticity, learning, and memory. Both loss-of-function and splice variants have been extensively studied and functionally characterized by Küry et al. in 2017 [31].

The *RALA* gene encodes a low molecular mass ras-like GTP-binding protein that shares about 50% similarity with other ras proteins. In neuronal culture systems, RALA has been implicated in the development, plasticity, polarization, migration, branching, and spine growth of neurons, as well as the renewal of synaptic vesicles and trafficking of NMDA, AMPA, and dopamine receptors to the postsynaptic membrane. Loss of function for *RALA* causes a severe neural tube defect; *de novo* missense variation disrupting the GTP/GDP-binding functions of *RALA* lead to developmental delay, intellectual disability, and related phenotypes (Hiatt-Neu-Cooper neurodevelopmental syndrome, MIM# 619311) [34].

The deletion of these genes could have influenced the phenotype of our patient, especially the intellectual status, but their impact is indistinguishable from the GCPS-CGS phenotype. The macrosomia, hypertelorism, prominent metopic suture without confirmed craniosynostosis, anomaly of the corpus callosum, and polysyndactyly can be attributed to Greig cephalopolysyndactyly syndrome and haploinsufficiency of GLI3. Intellectual deficiency is rare in loss-of-function mutations of *GLI3* associated with GCPS syndrome, but is frequently described and correlates with the size of deletion of GCPS-CGS [31].

Since the identification of *CDK13*, *CAMK2B* and *RALA* as syndromic, intellectual disability genes, we assume that the involvement of *CDK13*, *CAMK2B* and *RALA* genes in the deletion of our patient explains the presence and the severity of the intellectual disability. Callosal anomalies have also been reported in the *CAMK2B*-related intellectual disability [31].

To our knowledge, chromosomal deletion encompassing the genes *CDK13*, *CAMK2B* or *RALA* as a mechanism for intellectual disability related neurodevelopment delay disorders, have not been described so far.

Twelve further genes in the deleted segment: *NME8* (NME/NM23 Family; OMIM# 607421), *MPLKIP* (M-Phase-Specific Plk1-Interacting Protein; OMIM# 609188), *BLVRA* (Biliverdin Reductase A; OMIM# 109750), *PGAM2* (Phosphoglycerate Mutase 2; OMIM# 612931), *OGDH* (Oxoglutarate Dehydrogenase; OMIM# 613022), *ADCY1* (Adenylate Cyclase 1; OMIM# 103072), *DDC* (Dopa Decarboxylase; OMIM# 107930), *AEBP1* (Ae-Binding Protein 1; OMIM# 602981), *SFRP4* (Secreted Frizzled-Related Protein 4; OMIM# 606570), *PKD1L1* (Polycystin 1-Like; OMIM# 609721), *VPS41* (Subunit Of Hops Complex; OMIM# 605485), and *SUGCT* (Succinyl-Coa: Glutarate-Coa Transferase; OMIM# 609187) are related to recessive conditions, and the deletion of a single allele will not manifest as a disease unless the second allele has a mutation. The clinical signs of our patient cannot be correlated with the effects of these genes. Regarding the *DDC* gene, this would require further investigation (sequencing) given the risk of acute lymphoblastic leukemia (MIM# 613067) [14,35].

The *NPC1L1* (NPC-Like Intracellular Cholesterol Transporter 1; OMIM# 608010) gene is responsible for the intestinal absorption of cholesterol, it is the molecular target for the drug ezetimibe, and variants in this gene affect response to this drug. The inheritance of the trait is not known, but monitoring cholesterol profile is relatively easy.

We only assume that the patient’s deletion is *de novo* because, although the parents do not show clinical signs of the disease, it would have been necessary to perform q-PCR or microarray for them to exclude the inheritance of the deleted segment and high-resolution karyotype or FISH test to detect balanced chromosomal rearrangements. Genetic testing was declined by both parents, they are not willing to have further children.

A review of the literature of the patients with Greig cephalopolysyndactyly contiguous gene syndrome caused by a structural chromosomal anomaly (deletion) with a size greater than 1 Mb is summarized in Table 2 [3,4,6,7,11,13,16,17,18,19].

The genotype–phenotype correlation regarding the neurodevelopment in Greig cephalopolysyndactyly contiguous gene syndrome in our case and a further six cases (with available genomic coordinates) from the literature is presented in Table 3.

The haploinsufficiency of the *CDK13*, *CAMK2B* and *RALA* genes could influence the patients’ neurodevelopment in five cases and the *CAMK2B* gene in two cases.

## 5. Conclusions

The case presented here, with an 18 Mb deleted chromosomal segment, is the largest deletion described in the related literature so far.

By describing the multitude of symptoms in our patient related to this large spanning deletion, encompassing several other genes than *GLI3* responsible for intellectual disability, and by comparing our patient’s features with literature data, we aimed to broaden the phenotypic and genotypic spectrum of GPS-CGS and find out about the possible comorbidities and prognosis. Sequence analysis would be useful to determine whether the variants on the other allele could contribute to the phenotype, therefore a whole exome sequencing has yet to be performed.

## Figures and Tables

**Table 1:** Results of the cytogenetic- and the molecular cytogenetic investigations.

	Results	Size of the Deleted Segment (Mb)	OMIM Genes
Karyotype	46,XY, del (7)(p13-p15)		
aCGH	arr[hg38]7p14.2-p11.2 (35830920-54201451)x1	18.04	*ANLN*, *NME8*, *POU6F2*, *MPLKIP*, *GLI3*, *BLVRA*, *GCK*, *NPC1L1*, *OGDH*, *CCM2*, *ADCY1*, *IKZF1*, *DDC*, *PGAM2*, *CDK13*, *AEBP1*, *CAMK2B*, *SFRP4*, *PKD1L1*, *VPS41*, *SUGCT*, *RALA*
LOH	-	18.37	*ANLN*, *NME8*, *POU6F2*, *MPLKIP*, *GLI3*, *BLVRA*, *PGAM2*, *GCK*, *NPC1L1*, *OGDH*, *CCM2*, *ADCY1*, *IKZF1*, *DDC*, *CDK13*, *AEBP1*, *CAMK2B*, *SFRP4*, *PKD1L1*, *VPS41*, *SUGCT*, *RALA*, *SEPTIN7*

aCGH: comparative genomic hibridisation; a: array; arr: array; hg38: human genome version 38; LOH: loss of heterozygosity; Mb: megabase.

**Table 2:** Review of literature of the patients with GCP-CGS due to a chromosomal deletion encompassing *GLI3* (>1 Mb).

References	Chromosomal Localisation	Size of the Deleted Segment (Mb)	Array Coordinates
Present case	7p14.2-p11.2	18.37	arr[hg38](35830920_54201451)del
Niida Y. et al [3] (2015)	7p14.1-p12.3	6.2	arr[hg19](41076615_47282889)del
Demurger F. et al. [13] (2015)	7p13-p15	7	arr[hg19](38521704_45810267)del
		9	arr[hg19](35674000_37280000) _(46116000_46598000)del
Jane A Hurst et al [11] (2011)	7p13-p14.1	6.0	arr[hg18](39013006_39213707)del
		6.8	arr[hg18](39130081_45492392)del
	7p12.3-p14.1	8.3	arr[hg18](40845981_40855164) _(49136714_49160830)del
Solveig Schulz et a [4] (2008)	7p13-7p14	14	NA
Debeer Philippe et al. [16] (2007)	7p14.3	NA	NA
	7p14.3		
Jennifer J Johnston et al. [6,7] (2003, 2007)	7p14.1	1.8	NA
	7p14.1-7p13	3.2	
	7p14.1	4.1	
	7p14.1-7p13	5.2	
	7p14.1-7p13	5.9	
	7p14.2-7p14.1	6.3	
	7p14.1-7p12.3	8.4	
	7p14.2-7p13	9.8	
	7p14.2-7p13	10.3	
Kroisel PM et al [12] Schwarbraun T. et al. [18] (2001, resp. 2005)	7p13	4.5-15	NA
	7p12.3-p13		
	7p12.3-p13		
	7p12.3-p14.2		
	7p11.2-p13		
Williams PG et al. [17] (1997)	7p13-p15.1	NA	NA
Zneimer SM et al. [19] (2000)	45,XY,der(22;7) (p13;p22.3)del (7)-(p11.2-p15.1)	NA	NA

NA: not available.

**Table 3:** Genotype–phenotype correlation.

References	Patients’s Age	Size of the Deleted Segment (Mb)	Phenotype Associated OMIM Genes	Neurodevelopment
Present case *	3 years followed to age 6	18.37	*ANLN*, *NME8*, *POU6F2*, *MPLKIP*, *GLI3*, *BLVRA*, *GCK*, *NPC1L1*, *OGDH*, *CCM2*, *ADCY1*, *IKZF1*, *DDC*, *PGAM2*, *CDK13*, *AEBP1*, *CAMK2B*, *SFRP4*, *PKD1L1*, *VPS41*, *SUGCT*, *RALA*	Severe intellectual disability, speech and developmental delay
Niida Y. et al. * [3] (2015)	2 years	6.2	*GLI3*, *GCK*, *CCM2*, *AEBP1*, *CAMK2B*, *PGAM2*, *BLVRA*, *ADCY1*, *NPC1L1*, *OGDH*	Developmental delay
Demurger F. et al.* [13] (2015)	NA	7	*GLI3*, *AEBP1*, *MPLKIP*, *GCK*, *CCM2*, *CDK13*, *CAMK2B*, *VPS41*, *BLVRA*, *PGAM2*, *ADCY1*, *SUGCT*, *POU6F2*, *NPC1L1*, *RALA*, *OGDH*	Developmental delay
	NA	9	*GLI3*, *GCK*, *CDK13*, *MPLKIP AEBP1*, *CCM2*, *CAMK2B*, *SFRP4*, *VPS41*, *PGAM2*, *BLVRA*, *NME8*, *SUGCT*, *ADCY1*, *NPC1L1*, *POU6F2*, *ANLN*, *RALA*, *OGDH*	Developmental delay
Jane A Hurst et al.* [11] (2011)	2 years	6.0	*GLI3*, *GCK*, *CCM2*, *CDK13*, *MPLKIP*, *AEBP1*, *CAMK2B*, *BLVRA*, *PGAM2*, *SUGCT*, *RALA*, *POU6F2*, *NPC1L1*, *OGDH*	Developmental delay
	5 years	6.8	*GLI3*, *GCK*, *CCM2*, *CDK13*, *MPLKIP*, *AEBP1*, *CAMK2B*, *BLVRA*, *PGAM2*, *SUGCT*, *RALA*, *NPC1L1*, *POU6F2*, *OGDH*	Severe intellectual disability, speech and developmental delay
	15 years	8.3	*GLI3*, *GCK*, *CCM2*, *AEBP1*, *CAMK2B*, *PKD1L1*, *BLVRA*, *PGAM2*, *SUGCT*, *ADCY1*, *NPC1L1*, *OGDH*	Severe intellectual disability, speech and delayed development

*: updated cases based on the available array coordinates; Neurodevelopment disorders related genes: *CDK13*, *CAMK2B*, *RALA*.

## Data Availability

Not applicable.
